# An endoscopic ultrasonography-guided interstitial brachytherapy based special treatment-planning system for unresectable pancreatic cancer

**DOI:** 10.18632/oncotarget.15763

**Published:** 2017-02-27

**Authors:** Xiaotian Sun, Zheng Lu, Yijun Wu, Min Min, Yiliang Bi, Wei Shen, Yang Xu, Zhaoshen Li, Zhendong Jin, Yan Liu

**Affiliations:** ^1^ Department of Gastroenterology, the 307 Hospital of Academy of Military Medical Science, Beijing, China; ^2^ Department of Internal Medicine, Clinic of August First Film Studio, Beijing, China; ^3^ Liver Cirrhosis Diagnosis and Therapy Center, The 302 Military Hospital of PLA, Beijing, China; ^4^ Department of Electronic Engineering, Information Science and Engineering School, Fudan University, Shanghai, China; ^5^ Department of Gastroenterology, Changhai Hospital, the Second Military Medical University, Shanghai, China

**Keywords:** endoscopic ultrasonography, radioactive seeds, brachytherapy, pancreatic cancer, treatment planning system

## Abstract

EUS-guided interstitial brachytherapy is promising in the treatment of unresectable malignant carcinoma adjacent to the digestive tract. The feasible treatment plan is not established. Thus, our study aimed to develop a novel treatment plan and evaluate the feasibility in patients with unresectable pancreatic cancer. A total of 42 patients with unresectable pancreatic cancer (stage III: *n* = 18; stage IV: *n* = 24) were retrospectively included. A special treatment-planning system (TPS) for EUS was designed and evaluated by comparing with the traditional TPS. The patients underwent EUS-guided interstitial brachytherapy based on the new software. In the test model, there was no obvious difference of irradiation doses calculated by the two softwares (EUS TPS vs. traditional TPS) (*P* > 0.05). Under the support of EUS TPS, a novel treatment plan for EUS-guided interstitial brachytherapy was successfully established, which contained seven principles. All patients tolerated the treatment well without any serious complications. In 15 patients (stage III) whose minimal peripheral dose was larger than 90 Gy, partial remission rate was 80% (12/15). Twelve patients (12/18) in stage III were alive for over 12 months with a median peripheral dose of 107.5 Gy. The expected median survival time of the 42 patients was 9.0 months (95%CI 7.6-10.4 months). The results demonstrated that the new EUS TPS will play an important role in EUS-guided interstitial brachytherapy in patients with unresectable pancreatic malignant cancer.

## INTRODUCTION

Endoscopic ultrasonography (EUS)-guided interstitial brachytherapy has been carried out for therapy of malignant tumors. For some unresectable malignant lesions adjacent to the digestive tract, such as pancreatic cancer and local recurrence of rectal cancer, EUS-guided interstitial brachytherapy has some unique advantages. It has been accepted that EUS-guided puncture has the advantages of accurate positioning, mild injury, and shorter puncture distance than routine ultrasound and CT [1]. EUS-guided brachytherapy in patients with recurrent esophageal cancer in the perigastric lymph nodes has been reported [2, 3]. The two currently available clinical trials of EUS-guided brachytherapy in patients with pancreatic cancer came from China, including our group [4, 5]. This technique does not significantly improve the overall survival rate; partial remission (PR) rates of 27% and 13.6% were achieved in the two trials. Such disappointing results might be related to a variety of factors, one of which is the insufficient radiation dose to local lesions.

In conventional interstitial brachytherapy, a treatment-planning system (TPS) that can calculate the dose distribution is required. To date, there has been no mature TPS based on EUS imaging for clinical application. The number of radioactive seeds of EUS-guided interstitial brachytherapy is generally calculated by the modified Harila formula, and the placement of the implanted radioactive seeds is dependent on the experience of the operating physician and the puncture difficulty [5]. In linear EUS-guided puncture, the change of puncture plane relies mainly on the rotation of the EUS probe *in situ*, and accurate calculation and adjustment of the distance between the two puncture planes is difficult. Moreover, the puncture paths were limited to a certain range in the proper EUS section. These difficulties do not exist in transrectal ultrasound (TRUS)-guided interstitial brachytherapy in prostate cancer [6]. The irregular arrangement of radioactive seeds in the three-dimensional space and the current lack of special TPS might cause the development of a cold area (an area in which the local dose is less than the therapeutic dose), resulting in treatment failure. In our study, a type of computer-aided TPS based on EUS imaging was designed. This system can simulate seed implantation and calculate the dose distribution in the EUS section in real time. Moreover, the dose distribution in the three-dimensional space can be calculated. With the help of this software, a novel treatment plan of EUS-guided interstitial brachytherapy has been designed. Therefore, the aim of the current study was to evaluate the feasibility and efficacy of the new EUS TPS and its clinical application in unresectable pancreatic cancer.

## RESULTS

### Software design and verification

In the test model, the four radioactive seeds were arranged at the four vertices of a square with 1-cm side lengths. The absorbed doses at the points of 1 cm outside of the seeds in the plane were calculated. When the parallel movement of the plane in its vertical direction was 1 cm, the changes in the dose distribution at these points were also recorded. The irradiation doses at the points calculated by the two softwares (EUS TPS vs. traditional TPS) were compared in Table 1. There was no obvious difference between the calculation results (P > 0.05).

**Table 1:** Comparison of irradiation dose at certain points calculated by two TPS strategies.

Seeds activity, mCi	Vertical movement from the original position, cm	Irradiation dose calculated by EUS TPS, Gy	Irradiation dose calculated by commercial TPS, Gy	*P* value
0.5	0	22.9 ± 0.3	22.2 ± 0.4	0.271
	1	12.5 ± 0.1	12.5 ± 0.2	0.881
0.6	0	26.3 ± 0.4	26.6 ± 0.5	0.278
	1	14.9 ± 0.1	15.1 ± 0.1	0.108
0.7	0	30.9 ± 0.8	30.8 ± 0.3	0.885
	1	17.6 ± 0.2	17.5 ± 0.1	0.464
0.8	0	35.3 ± 0.9	35.2 ± 0.3	0.683
	1	20.0 ± 0.3	20.0 ± 0.1	0.873

### Treatment plan of EUS-guided interstitial brachytherapy

#### Dose calculation in puncture plane

**Figure 1:**
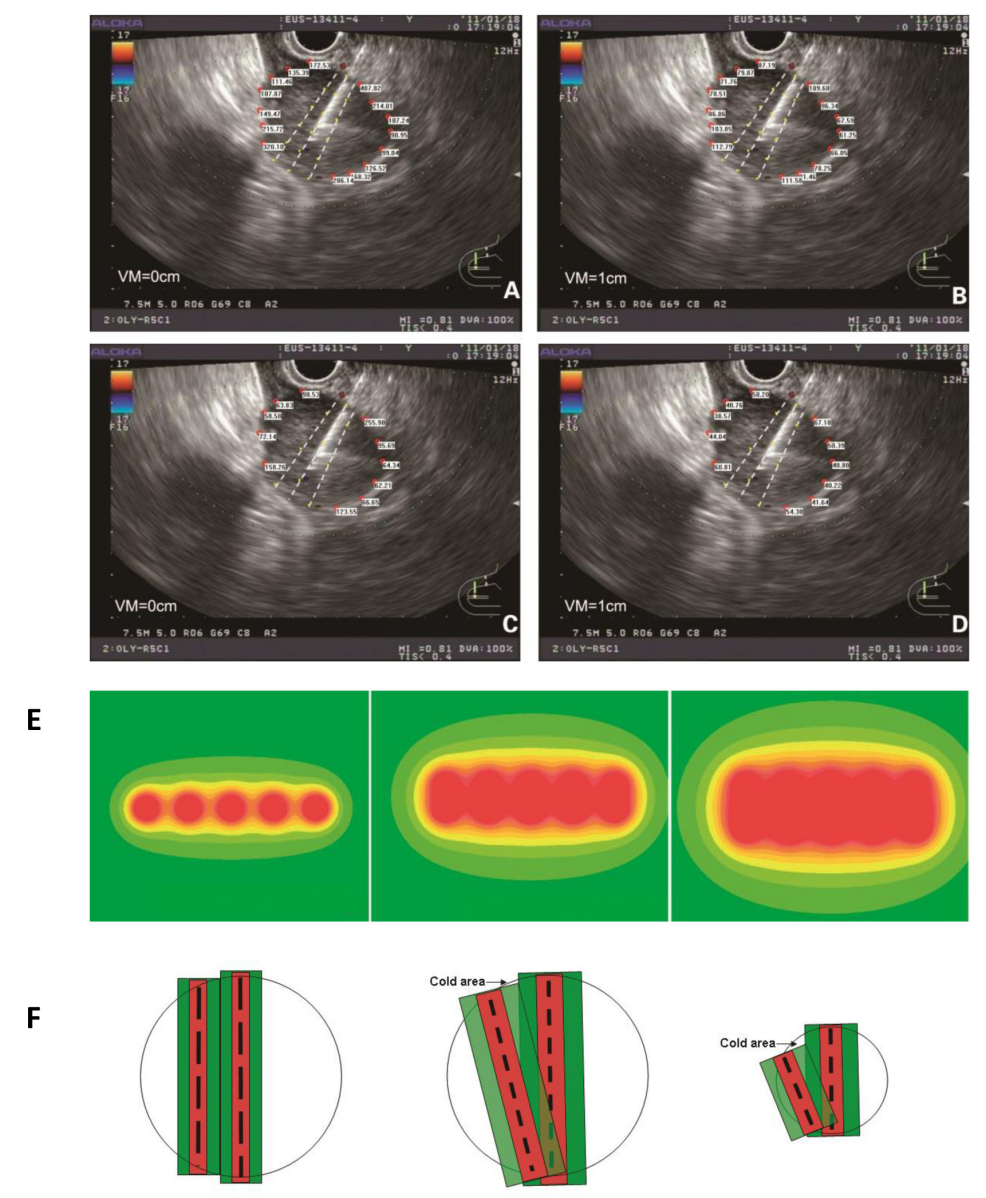
The peripheral dose distribution at the edge of the tumor was calculated at 5-mm interval **(A),** and the change in the dose distribution after 10-mm vertical movement were also detected **(B).** The peripheral dose distribution at the edge of the tumor was calculated at 10-mm interval (**C.**) with the change in the dose distribution after 10-mm vertical movement (**D.**) **E.** The peripheral dose distribution of a radioactive seed chain (5 seeds, 5-mm interval) (left), of two parallel radioactive seed chains (5 seeds, 5-mm interval) (middle) and of three parallel radioactive seed chains (5 seeds, 5-mm interval) (right) indicated that in accordance with the descending order of doses, the color changes gradually from red to yellow. Local irradiation doses <90 Gy were shown as a green coloration. **F.** The dose distribution between two parallel puncture planes under traditional prostate brachytherapy (left). The cold area of dose distribution between two puncture planes under EUS-guided brachytherapy (middle). The long black dotted line represents the radioactive seed chain at 10-mm interval. The short black dotted line represents the radioactive seed chain at 5-mm interval. The absorbed dose was greater than the therapeutic dose in red area. And in green area, the absorbed dose was between the therapeutic dose and 1/2 therapeutic dose. When the tumor was larger in diameter, the cold area (black arrow) was not obvious under the small rotation angle. When the tumor diameter was small, the cold area (black arrow) was obvious (right). VM: vertical movement.

At the same length, radioactive seed chains at 10-mm interval had a smaller radius of irradiation than those at 5-mm interval, which necessitated the implantation of more seeds (Figure 1A-D). If the lateral edges of the tumor were not close to the target puncture area, an increase in the puncture time was considered and more chains of radioactive seeds were implanted within the target area, thereby expanding the irradiation area. There was high dose distribution within the target puncture area, which differed from the dose pattern of the traditional arrangement at 1-cm interval (Figure 1E). The puncture target area should be as close as possible to the central part of the tumor in the EUS section, to avoid radiation-related complications. Based on the TPS software calculation, the minimal peripheral dose for the tumor should not be less than the therapeutic dose.

#### Dose calculation in space

After the calculation, when both two conditions are satisfied: (1) the spacing between the radioactive seeds was 5mm; (2) the minimal peripheral dose was not less than the therapeutic dose, the appropriate length of H was about 1 cm. The rotation angle of the EUS probe (α) could be calculated by the following approximation formula: α= 115/ R.

#### Modification of the implantation program

**Figure 2:**
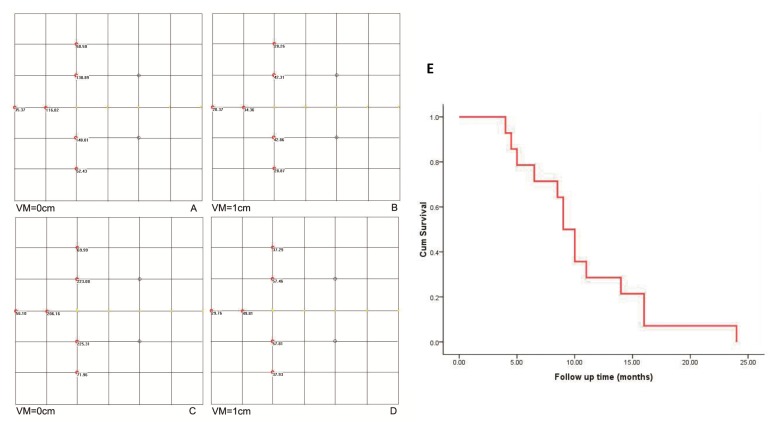
The peripheral dose distribution of a radioactive seed chain (0.5 mCi, 5 seeds, 5-mm interval) (**A**) with the changes in the dose distribution after 1-cm vertical movement (**B**) were shown**.** The peripheral dose distribution of an extra seed placed on the end of a radioactive seed chain (**C.**,) and the change in the dose distribution after 1-cm vertical moment (**D.**) were also manifested. The seeds were shown in the image as yellow dots. **E.** Cumulative survival curve of 84 patients with pancreatic cancer. VM: vertical movement.

According to the above implantation model, when H=1 cm was used, there was a significant dose-insufficient cold area near the tumor edges distal from the EUS probe. The target areas in two adjacent puncture sections before and after probe rotation were different, which caused the cold region. This issue was absent from the traditional implantation model with 1-cm intervals between two parallel sections (Figure 1F). Two strategies were employed to overcome this problem. One involved increasing the puncture depth in the EUS section with a smaller target area, which was associated with some technical risk. The other method involved placement of additional radioactive seeds at the edge of the tumor distal from EUS probe. The distance between the adjacent aspirations increased gradually due to the fan-shaped distribution. This method not only effectively compensated for the non-uniform dose distribution at the distal edge, but also expanded the irradiation range and overcame the dose insufficiency in the cold area. There was no increase in the number of punctures or in the operational risk (Figure 2A-2D).

The principles of treatment plan were selected as following: a) The procedure is mainly based on the rotation of the EUS probe *in situ*. If the tumor size is too large, the tumor will be divided into several “separate” targets by the forward-backward or up-down movement (not rotating movement). b) In general, the maximum cross-section of tumor is selected as the first puncture plane and the maximum distance (R) from EUS probe to tumor edge in this section is measured. c) The puncture target area should be as close as possible to the central part of the tumor in the EUS section. d) The optimum spacing between the radioactive seeds is 5 mm and the single activity is 0.8 mCi. e) The minimal peripheral dose in every puncture section should not be lower than the therapeutic dose. f) The vertical movement (H) is calculated when the minimum peripheral doses at the corresponding point of EUS section reach half the therapeutic dose. When the conditions d) and e) are satisfied, the rotation angle (α) of EUS probe can be calculated by the approximation formula: α= 115/ R. g) The additional radioactive seeds should be placed at the edge of the tumor distal from EUS probe.

#### Experimental results on clinical application of TPS

**Figure 3:**
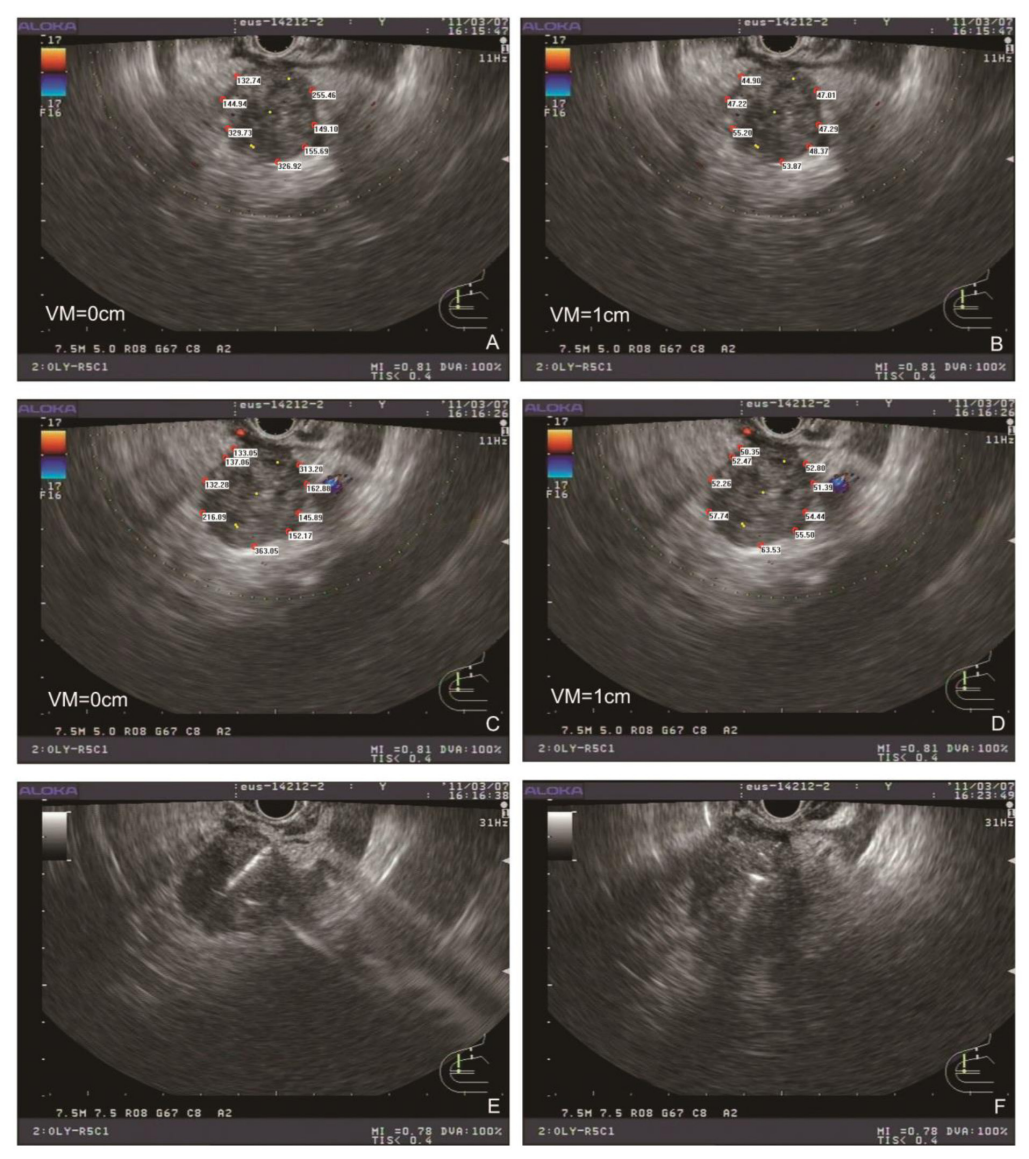
Typical simplified TPS and EUS-guided puncture procedure The peripheral dose distribution in maximal EUS cross section (**A**) with the change in the dose distribution after 1-cm vertical movement (**B**). The seeds were shown as yellow dots. The peripheral dose distribution after rotation of the EUS probe (**C**) with the change in the dose distribution after 1-cm vertical movement (**D**). Radioactive seeds were placed under EUS-guided puncture (**E**), and EUS section after the placement of radioactive seeds were recorded (**F**). VM: vertical movement.

**Figure 4:**
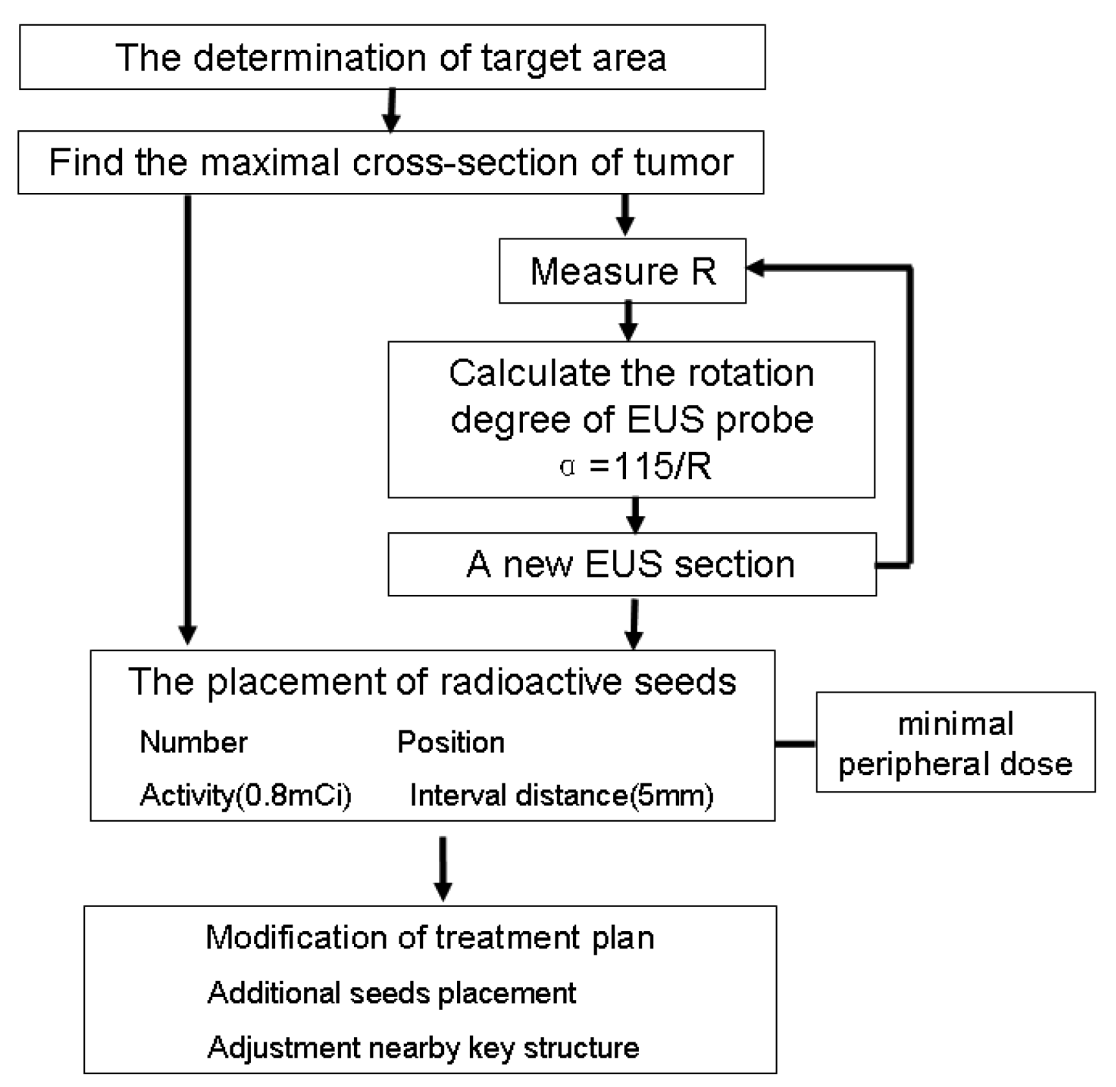
Diagram of the general TPS protocol of EUS-guided brachytherapy for unresectable pancreatic cancer

All patients generally tolerated the treatment well without any serious complications throughout the study. Of the 42 patients, 36 were treated with only one implantation, and 6 underwent implantation twice. The average number of seeds (0.8 mCi) implanted was 22 per patient (range 10-35 per patient). The average minimum peripheral dose at the edge of the tumor was 94.9 ± 20.1 Gy (54.2-140.6 Gy). All patients were followed up for a median of 10.5 months (range 4-24 months). At 2 month after the first seed implantation, the rates of complete remission (CR) and PR, stable disease (SD), and partial development in the 18 patients of stage III were 0% (*n* = 0), 66.7% (n = 12), 16.7% (*n* = 3), and 16.7% (*n* = 3), respectively. In 15 patients that the minimal peripheral dose was larger than 90 Gy, the PR rate was 80% (12/15). Thirty-three patients were totally followed up for more than 6 months, and 12 patients (12/18) in stage III were alive for more than 12 months with a median peripheral dose of 107.5 Gy. No significant difference in the serum CA19-9 level was found before and 1 and 3 months after brachytherapy. According to the Kaplan-Meier analysis, the expected median survival time of the 42 patients was 9.0 months (95% confidence interval [CI] 7.6-10.4 months) (Figure 2E). Figure 3 showed a simplified treatment plan protocol and EUS-guided puncture procedure. In the 6 patients with pancreatic head carcinoma, the number of passages under EUS as well as the actual number of seeds implanted was lower than that predicted by the TPS system because of puncture difficulty. Taken together, the general protocol of EUS-guided interstitial brachytherapy were seen in Figure 4.

## DISCUSSION

Interstitial brachytherapy has been considered a useful method for the local control of malignant tumors. Clinically, the technique has also been used to control malignancies of the prostate, breast, brain and rectum [7, 8]. After radioactive seed placement, the target tissue is continuously exposed to γ-rays, which produces more localized tissue injury and tumor ablation than intraluminal brachytherapy. Intraluminal and interstitial brachytherapies have been found effective when used as palliative therapy to improve local control in patients with locally advanced pancreatic cancer. It has been reported that intraluminal brachytherapy from the duct of Wirsung and the common bile duct are safe and feasible for the treatment of pancreatic head carcinoma [9]. Peretz et al. used visible implantation of 125I seeds during surgery to treat 98 patients with pancreatic cancer, and produced a response rate of 45% and a pain relief rate of 65% [10]. Feasibility of this approach has also been confirmed by the previous clinical studies [5]. However, the conventional methods of implantation, which are generally performed either under open surgery or by guided imaging techniques (e.g. CT), have some disadvantages.

TPS was necessary to create an interstitial brachytherapy treatment plan. The number, activity, and position of the radioactive seeds in the tumor were calculated by TPS, and the peripheral dose at the edge of the tumor was determined [11]. The arrangement of the seeds calculated by traditional TPS was regular with equal distances along parallel straight lines. The adjacent radioactive seeds were arranged as a square or equilateral triangle [12]. Under EUS, the cross section of the tumor was determined by real-time sector ultrasound, and the relationship between the surrounding vascular system and the tumor was identified. The puncture paths should be determined by color Doppler technology to prevent injury to the pancreatic duct or the vessels. The radioactive seeds were placed along the puncture paths at equal distances, while the paths were usually not parallel. It is difficult to calculate the dose distribution using traditional TPS. With the help of the new EUS TPS, the dose at any point in the puncture section can be calculated according to the seed number and single activity. In our study, a traditional TPS model with a regular radioactive seed arrangement was selected. There was no obvious difference between the absorbed doses at the points calculated by the new TPS and the commercial TPS. The accuracy of the new TPS was thus confirmed. Therefore, in three-dimensional space, the arrangement of radioactive seeds was irregular [5, 13, 14]. At present, the dose calculation at any point on the three-dimensional edge of the tumor, which was not in the puncture section, is difficult. An additional function was designed in the new EUS TPS. The change in the dose distribution at the edge of the tumor could be calculated in real time when the EUS section moved in the vertical direction. In our study, a novel EUS-guided brachytherapy treatment plan was designed using this function.

In the traditional treatment plan, the distance between two parallel layers of radioactive seeds was about 1 cm [15]. However, in the new puncture mode, if the maximum distance between the two non-parallel puncture surfaces was set at 1 cm, the puncture times and complications would increase greatly. If the minimum peripheral doses on a puncture surface using 5-mm-spacing radioactive chains was not less than the therapeutic dose (≥90 Gy), the vertical movement of the plane was about 1 cm when the irradiation dose at the corresponding point reached half the therapeutic dose. The maximum distance between the two non-parallel layers would then reach about 2 cm. In the interstitial brachytherapy of pancreatic cancer, the best recommended therapeutic dose is not yet clear. NCCN recommends the adjuvant radiation therapy dose is 45-54 Gy after surgical removal of the tumor. For unresectable pancreatic carcinoma, the recommended radiation dose is 50-60Gy. A dose of 110 to 160 Gy is recommended for good local control in intraoperative interstitial brachytherapy of pancreatic cancer [16, 17]. In our study, the minimal peripheral dose at the edge of the tumor in 36 patients was >90 Gy. It was difficult to implant a sufficient number of seeds in 6 cases of pancreatic head carcinoma, resulting in a non-therapeutic dose and a poor prognosis. In cases of procedural difficulty, new approaches should be developed to improve the puncture technique. In some cases, if the tumor is large or achieving an adequate treatment dose at the tumor edge is problematic, the effects of treatment may be less than ideal. In these situations, external beam radiotherapy or external beam radiotherapy combined with brachytherapy should be used. In the treatment of prostate cancer, when the external radiotherapy combined, the proper dose was selected as 40-50 Gy, the irradiation dose of brachytherapy was adjusted to 100-110 Gy (recommended therapeutic dose 144 Gy) [18].

In our study, the expected median survival time of the 42 patients was 9.0 months. However, 24 patients were in stage IV. In these patients, EUS-guided brachytherapy was performed only for local treatment, and should be combined with chemotherapy and other methods. Fifteen of 18 patients in stage III received a dose greater than 90 Gy. The average survival time was 16.2 months, and 12 patients achieved PR, demonstrating encouraging results. The patients with a minimum peripheral dose of more than 90 Gy achieved a good treatment effect, but the treatment effect in the other patients who received a low peripheral dose was poor. Therefore, assurance of an adequate peripheral dose is important for a successful treatment outcome.

In conclusion, our data suggest that the new TPS based on EUS images can calculate the dose distribution in EUS section with an interactive interface. This software will play an important role in EUS-guided interstitial brachytherapy in patients with unresectable pancreatic malignant carcinoma. However, the present study has some limitations, including its non-case-controlled nature and the small number of patients recruited. Therefore, further prospective studies of an appropriate design and adequate sample size are necessary to evaluate the new software.

## MATERIALS AND METHODS

### Software design and verification

**Figure 5:**
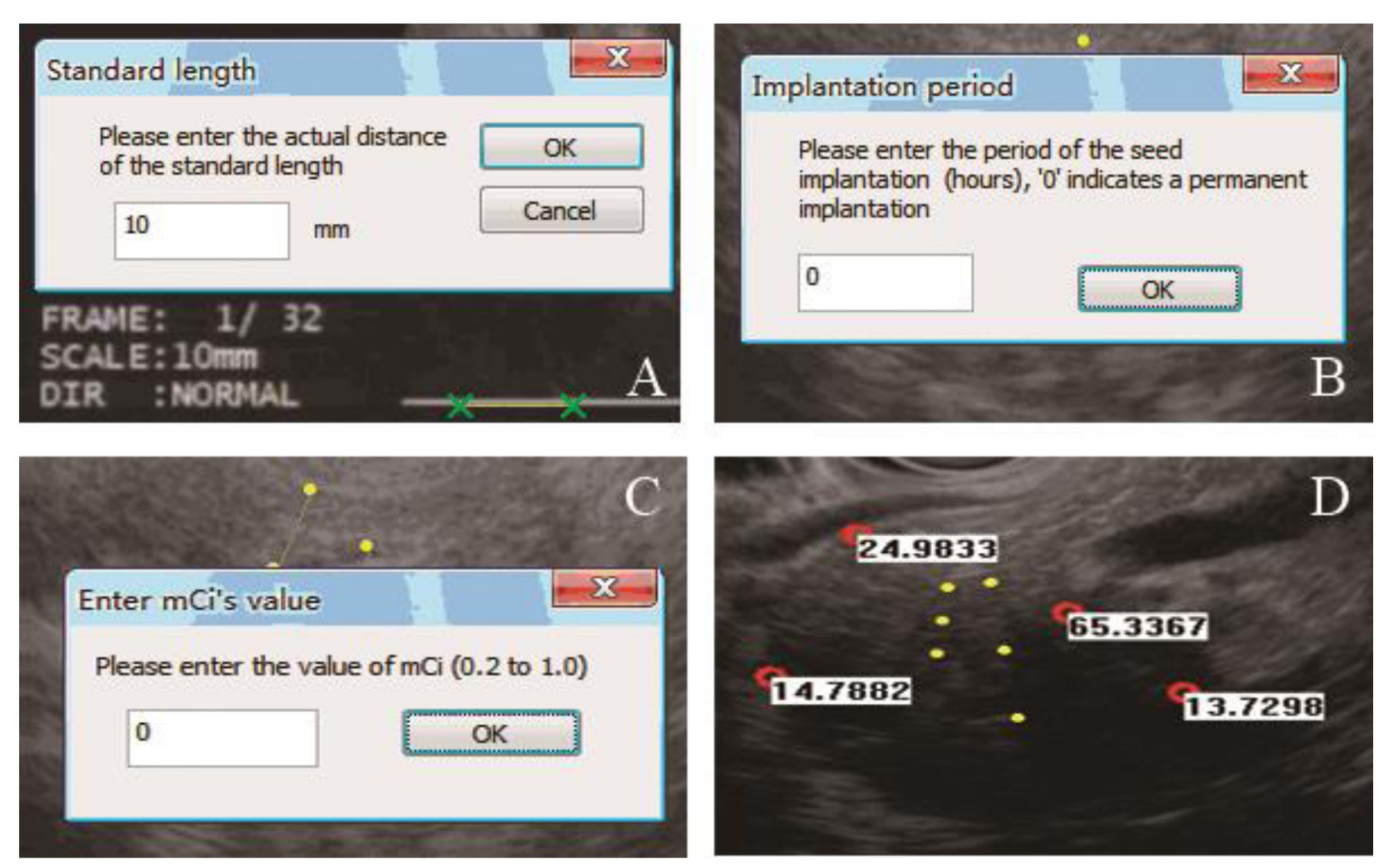
EUS TPS procedures were shown **A.** The standard length was entered according to the actual length on the image. **B.** The period of seed implantation (hours) was entered in this window. **C.** The single activity of a radioactive seed was entered. **D.** The planted seeds are shown in the image as yellow dots. The absorbed radiation dose (*Gy*) is shown beside each selected point (red dots).

The new software of EUS TPS had the same principle with traditional TPS, which were both developed on the basis of the AAPM TG 43 [19]. Some program designs had been adjusted to accommodate the new clinical requirements in the new software. In EUS TPS, the radioactive seed was selected as iodine 125 seeds (6711 type). The detailed process of the EUS TPS was shown in Figure 5. First, the standard length was entered according to the actual length in the EUS image. The start and end points in the puncture path were then selected. A dialog box appeared, and the interval between each seed could be chosen (5 or 10 mm). The implanted seeds were then shown on the image as yellow dots. After simulation of seed implantation, the points of interest could be selected on the image (red circles). The values of the implanted period and single activity of radioactive seed (*mCi*) were entered. Next, the absorbed radiation doses (*Gy*) were calculated at the selected points. The positions of the seeds could be deleted or changed. The implanted period and *mCi* could also be altered at any time. When the EUS puncture section moved in the vertical direction, the change in the dose distribution in this section could be calculated in real time.

A common arrangement model of radioactive seeds in the clinical setting was selected to assess the accuracy of the new TPS. Four iodine 125 seeds were distributed at the 4 vertices of a square with 1-cm side lengths. The absorbed doses at the points of 1 cm outside of the seeds in the plane were calculated. When the parallel movement of the plane in its vertical direction was 1 cm, the changes in the dose distribution at these points were recorded. The absorbed dose calculated by the new TPS was compared with that from a commercial TPS approved by the Chinese State Food and Drug Administration (Kelinzhong Institute of Atomic Energy, Beijing, China; No. YZB/1466-70-2004). The process was repeated three times.

### Treatment plan of EUS-guided interstitial brachytherapy

#### Determination of target area

The morphology and location of the tumor were scanned by EUS carefully, and the maximum cross-section of the tumor was determined. The maximum and minimum diameters of the tumor were measured, and the important structures within and around the target were marked. In general, the maximum cross-section was first chosen to implant the radioactive seeds.

#### Dose calculation in puncture plane

**Figure 6:**
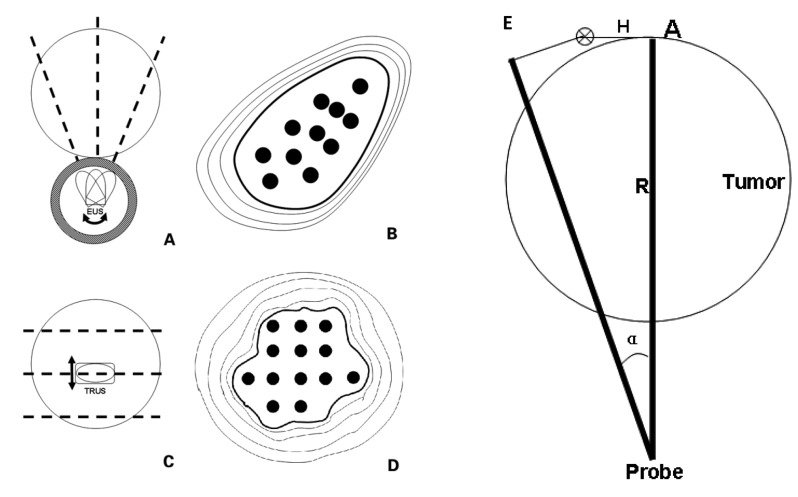
Differences were observed between EUS-guided puncture and traditional prostate brachytherapy, including rotation transformation of puncture section under EUS-guided brachytherapy **(A.),** irregular arrangement of radioactive seeds in an EUS section (**B.**), parallel transformation of puncture section under traditional prostate brachytherapy (**C.**), and regular arrangement of radioactive seeds in an section (**D.**) The black dotted line represented the puncture section. The radioactive seeds (black dots) were arranged at equal distances along straight lines. **E.** Transformation of puncture plane was through a rotation (α^0^ Degree) of the EUS probe. R was the distance from the point A to EUS probe. H indicated the vertical movement when the minimum peripheral doses at point A reached half the therapeutic dose. The target tumor was assumed to be a spherical mass proximal to the digestive tract.

In the process of EUS-guided brachytherapy, the transformation of the puncture section was achieved principally through rotation of the EUS probe. In the appropriate puncturing plane, a target puncture area was determined, which was positioned at about 6 to 7 o’clock on the EUS image. In contrast to other implantation methods, the radioactive seeds could not be implanted in the region outside the target area through EUS guidance. The radioactive seeds were arranged along the puncture path at a certain interval. Assuming the target tumor was a spherical mass close to the digestive tract, the differences between EUS-guided puncture and traditional prostate brachytherapy are shown in Figure 6A-D. The absorbed doses at the edge of the tumor on the EUS image were calculated, and the lowest value was defined as the minimal peripheral dose. The proper activity of a single seed and the seed arrangement were calculated to ensure that the minimal peripheral dose was not lower than the therapeutic dose.

#### Dose calculation in space

The moving distance of the EUS puncture section in the vertical direction was recorded when the minimal peripheral dose reached half of the therapeutic dose. The rotation angle of the EUS probe was then calculated according to the following modified formula (Figure 6E): α=H × 360/πR, where R was the maximum distance from the probe to the tumor edge in proper EUS section, the vertical movement of the plane was H when the minimum peripheral doses reached half the therapeutic dose at the corresponding point. An excessive rotation angle would result in a wide cold area with an insufficient radiation dose. However, the angle of rotation should not be too small, which would increase the time of punctures and the risk of complications.

#### Modification of the treatment plan

The region of the cold area in which the cumulative absorbed dose was less than the therapeutic dose was calculated. Additional radioactive seeds should be replanted within or near the cold area to supply the necessary radiation. Therefore, the absorbed dose in the important anatomical structures around the target area, such as the pancreatic duct and blood vessels, could be calculated. The local distribution and amount of radioactive seeds were adjusted to avoid irradiation complications.

### Clinical application of the treatment plan

Patients treated by any previous irradiation or a previous course of chemotherapy were excluded. Abdominal pain and other accompanying diseases were required to be controlled in all patients before inclusion in the study. While receiving implantation treatment, the patients also received other necessary treatments such as chemotherapy or biological therapy. The procedure was similar to our previous report [5]. The proper puncture section was determined mainly by the rotation of the EUS probe. When the tip of the needle reached the edge of the tumor distal from the probe, an appropriate number of radioactive seeds were placed in the area without withdrawing the puncture needle. The radioactive seeds were then pushed out as a line at an appropriate interval while the needle was withdrawn to the appropriate distance. In the treatment plan, the therapeutic dose was set at 90 Gy according to the previous literatures [20-23].

### Patients and follow-up

From November 2010 to November 2014, a total of 42 patients were enrolled. There were 24 males and 18 females with a median age of 72 years (range 54-86), and the median Karnofsky performance status (KPS) score was 70 (range 50-90). Of these patients, 6 had a tumor in the pancreatic head and 36 had a tumor in the pancreatic body or tail. Eighteen patients were in stage III, and the remaining patients were in stage IV with liver metastasis. Table 2 shows the details of the tumor location, size, and follow-up data of the 42 patients. Patient eligibility criteria included pancreatic adenocarcinoma histologically confirmed either unsuitable for surgical resection. To be included in the study, patients were required to have a KPS score of ≥ 50 and were expected to survive for more than 3 months after diagnosis; they were also required to have adequate bone marrow function (blood leukocytes ≥ 3.0 × 10^9^ cells/L, platelet count ≥ 100 × 10^9^/L, and hemoglobin ≥ 100 g/L). Patients with a prothrombin time of 3 s longer than the control were excluded. Written informed consent was required from all patients, and the study was approved by the institution’s ethics committee. All 42 patients entered the follow-up phase immediately after the first implantation. The follow-up visits were at 1 week, 1 month, 3 months, and every 3 months until 12 months. The tumor diameter and general condition of patients were monitored and recorded during follow-up. The short-term efficacy was determined according to the tumor response standards suggested by the World Health Organization [24]. The long-term efficacy included the median survival time and 1-year survival rate.

**Table 2:** Clinical data at baseline and treatment effect.

	Patients (*n*=42)
Median age (range), years	72 (54–86)
Male: female, n	24:18
Location of tumor, n	
Head of pancreas	6
Body or tail of pancreas	36
Clinical stage, n	
III	18
IV	24
Median KPS score (range)	70 (50–90)
Diameter of tumor under EUS, Mean ± standard deviation, cm	3.13 ± 1.62
Median follow-up period (range), months	10.5 (4–24)
Treatment effect	
CR	0
PR	12
SD	21
Progressive disease	9
Serious complications	0

### Statistical analysis

A paired *t*-test or non-parametric test was used to analyze numerical data between groups. The median survival time was evaluated by the Wilcoxon test and Kaplan-Meier method. Statistical analyses were performed using the Statistical Package for the Social Sciences software (SPSS version 10; SPSS Inc., Chicago, IL). Results were considered statistically significant at *P <* 0.05.
